# Tracheotomy for Foreign Bodies in the Air Passages*Read before the Atlanta Society of Medicine, at its regular meeting, Sept. 9th, 1890.

**Published:** 1890-10

**Authors:** W. F. Westmoreland

**Affiliations:** Professor of the Principal and Practice of Surgery and Clinical Surgery, Atlanta Medical College


VOL. VII. OCTOBER, 1890. No. 8.

### Original Communications.

### TRACHEOTOMY FOR FOREIGN BODIES IN THE AIR PASSAGES.*

By W. F. WESTMORELAND,

Professor of the Principal and Practice of Surgery and Clinical Surgery, Atlanta Medical College.

My attention has been particularly called to this subject by several cases I have had to operate on in the last few weeks.

These cases, while not common, occur frequently enough to deserve more than passing notice from the surgeon, who may be called upon to operate on them.

As Gross has observed, no man, however old or however great his opportunities for observation, can possibly have much experience in this branch of surgery. It therefore follows that any doctrine (rule) as to the handling of these cases must rest upon the united experience of the profession, rather than upon any one's personal dicta.

The only way to study satisfactorily the collected experience of the profession is as it is brought together in statistical tables.

From the study of such tables, and my own personal experience in the treatment of these cases, I have laid down this rule for my guidance, that, the certainty of the presence of a foreign body in the air passages makes an operation immediately necessary, and from this I should make an exception only in one class of cases, those when the foreign body had descended into one of the divisions of the bronchi.

A great variety of foreign bodies find their way into the air passages. The substance generally gets in during inhalation, or

*Read before the Atlanta Society of Medicine, at its regular meeting, Sept. 9th, 1890.

while laughing or coughing, and of course occurs as a rule amongst children. The symptoms exhibited by these cases, vary according to the situation in which the foreign body is lodged, and its nature; it may lodge in the ventricles, or if light, as a seed, and this is the most frequent substance that finds its way into the air passages, remain in the trachea and be carried up and down by the movements of respiration; or if it is heavy, as a coin, it is very apt to drop into one of the divisions of the bronchi, most frequently the right, and remain there. The symptoms will be more aggravated if it is irregular in shape or has sharp outlines.

The length of time that has elapsed since the occurrence of the accident also plays an important part in the seriousness of the symptoms.

The symptoms for convenience may be divided into three stages:

1. Obstruction immediately following the accident.
2. Irritation produced by its presence.
3. Inflammation coming on at a later period.

If the patient survives, the immediate results of obstruction, the stage of irritation follows this is the time that the patient, most frequently comes under the surgeon's notice. If nothing is done the period of inflammation gradually follows.

There is no marked difference in the symptoms of the three stages, and we will only speak of them in a general way.

In all cases there is a feeling of intense suffocation, great difficulty of breathing, violent fits of spasmodic coughing, frequently attended with vomiting. The patient's face is haggard and wears a look of great anxiety. These symptoms subside, and are followed by a few moments of rest, but any movement on the part of the patient brings them on with renewed violence.

Every time the foreign body is coughed up and strikes against the interior of the larynx it brings on the feeling of suffocation, and an attack of spasmodic coughing, and if it happens to become impacted there sudden death may result, apparently by the spasm induced. There have been a number of deaths from this before tracheotomy could be performed. I had a case that came very near dying in this way, and it convinced me that these cases do not bear waiting on, though the symptoms may be very mild. The case was that of a child two or three years old, in which the symptoms were unusually mild. The mother was sitting in the waiting room with the little one in her lap asleep. I opened the door, going into the room, when the youngster waked up and began to cough. In a moment he stopped breathing, and threw himself back making violent efforts at respiration. His face began to turn purple, and the mother began to shriek. I saw the situation was very critical, and that something had to be done quickly to dislodge the foreign body or the child would be dead in a few moments. I grabbed the child from the mother's arms, and rushed into the operating room, and in less time than it takes to write it I had pushed an English catheter through the glottis, and dislodged the body, and relieved the spasm. In a few minutes the child was breathing all right again. It is unnecessary to say that the child was operated on immediately, as they all will be in the future. I shall never forget the scene, though it lasted only a few moments. The look of anguish on the mother's face, or the look that she gave me, as she pressed wild-eyed close to the lounge, or the child when the body was displaced, losing its harrowing look of mortal agony, which was replaced by an expression that betokened relief and peace, and never will I again expose myself to a similar one, but shall prevent it by operating at once.

In making a diagnosis of these cases, I depend upon the history, that is, the sudden onset of symptoms, without previous history of any throat trouble, the fact that the patient thinks he has swallowed something, and principally upon hearing the whirr as the foreign body is carried up and down the trachea by the movements of respiration, and the click as it strikes against the walls or of the trachea or larynx. In operating on these cases it is not necessary to give an anæsthetic, but merely a matter of option, for as soon as the incision is made through the skin there is no pain; however I always give it as it keeps the patient perfectly quiet. Then by careful dissection, layer by layer, I go down to the trachea. When this is reached, I stop all hemorrhage, ligating every vessel, whether vein or artery, with cat-gut that bleeds before opening the trachea; when this is opened I put in a pair of tracheal forceps to keep it open. If the foreign body is not blown out, I let patient come out from under ether, and then excite enough irritation by movement of forceps to cause coughing, when the body generally comes out; if it does not I keep tracheal wound open until it does. When the body comes out, I wait a few minutes for mucus to be coughed out, then close the wound with three separate layers of continuous cat-gut sutures, the first through membrane enclosing the trachea, also the isthmus of thyroid gland, if it has been cut, second bringing the muscles together and the third for the skin. I leave a small opening at the lowest point of the wound for drainage, and then dress antiseptically.

I have had nine cases under my personal attention without an unfavorable result, nor do I think the percentage of deaths that statistics shows us occurs is at all necessary, if the operation was performed early instead of waiting until it is a dernier resort.

